# Cervical spine multiple myeloma and isolated radiotherapy

**DOI:** 10.1093/jscr/rjaa563

**Published:** 2021-01-18

**Authors:** Diogo Belo, José Hipólito Reis, Joaquim Cruz Teixeira

**Affiliations:** Neurosurgery Department, Centro Hospitalar Lisboa Norte (CHLN), Lisbon, Portugal; Neurosurgery Department, Centro Hospitalar Lisboa Norte (CHLN), Lisbon, Portugal; Neurosurgery Department, Hospital CUF Infante Santo (HCIS), Lisboa, Portugal

## Abstract

Multiple myeloma is a hematologic malignancy frequently presenting with spinal lytic lesions. The authors report the case of a patient with an extensively destructive osteolytic MM lesion in the cervical spine treated exclusively with radiotherapy. Computed tomography and magnetic resonance imaging scans showed an arrest of further progression of instability and resolution of the lytic lesion, showing signs of new bone formation. Whereas surgery should be considered for cases of spinal instability and potential neurological injury, this case demonstrates that isolated radiotherapy can be used in select cases to treat MM lesions and restore the structural integrity of the spinal elements.

## INTRODUCTION

Multiple myeloma is a hematologic malignancy in which clonal B-lymphocyte expansion occurs, frequently presenting with spinal osteolytic lesions [1].

Myeloma cells that invade the marrow spaces of the spine induce secretion of osteoclast-activating factors that promote osteolysis, often leading to axial pain due to compression fractures and spinal instability [3].

The first line of treatment is radiation therapy, as MM is known to be highly radiosensitive. However, in certain cases of potential neurological injury, such as spinal cord compression, a surgical approach should be considered [2, 5].

The authors report the case of an extremely destructive and unstable MM lesion in the cervical spine, with significant risk for devastating neurological injury, treated solely with radiotherapy.

The results of isolated radiation therapy for multiple myeloma metastases in the cervical spine with documented biomechanical instability have been scarcely reported.

## CASE REPORT

A 73-year-old man presented to our Department with cervical and right arm pain in the C5 and C6 dermatomes, C5 motor deficit (proximal and distal motor power grade II) and ipsilateral Hoffman’s. Physical examination was otherwise unremarkable.

**Figure 1:**
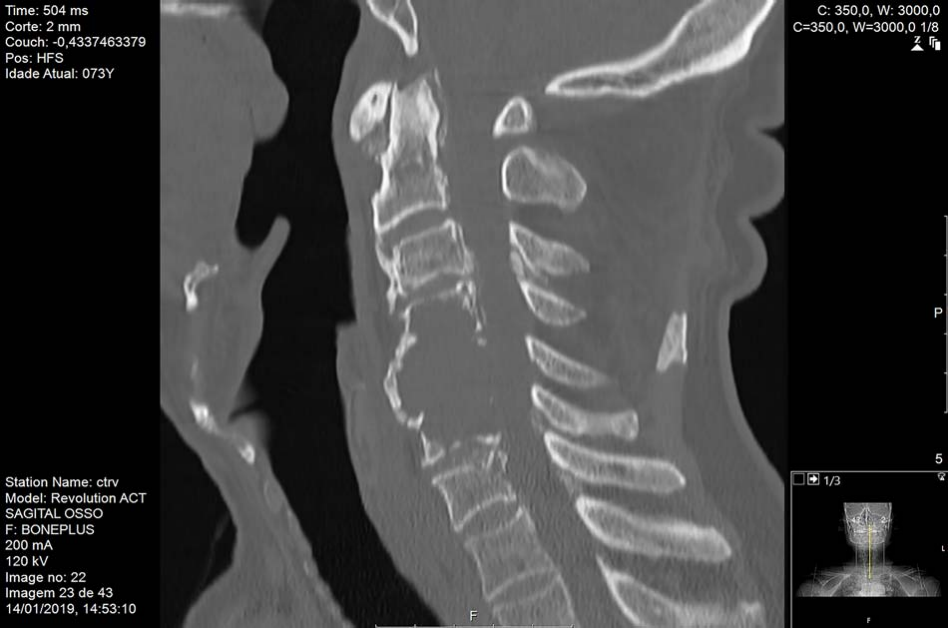
CT Sagittal pre-RT.

**Figure 2:**
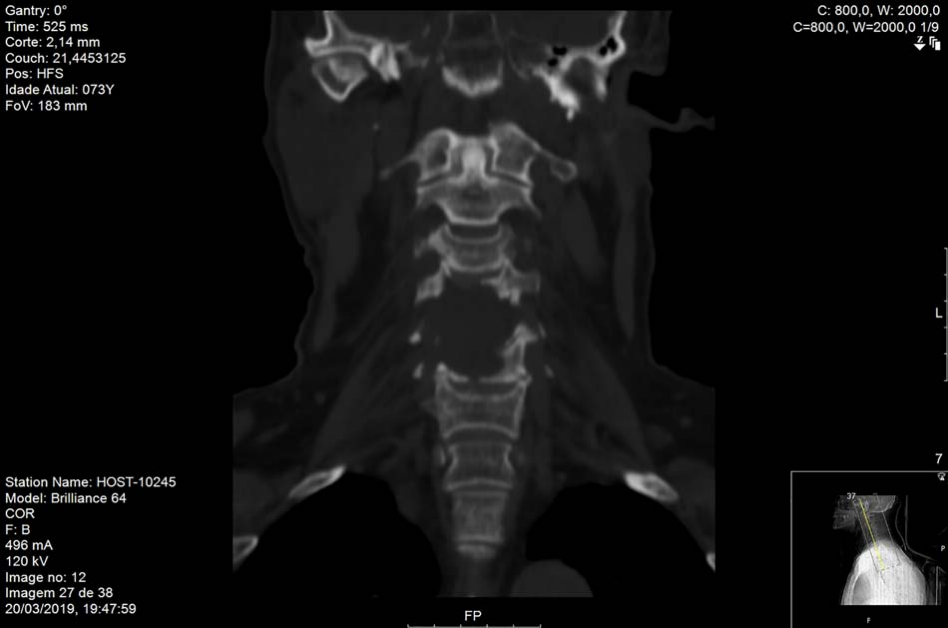
CT coronal pre-RT.

**Figure 3:**
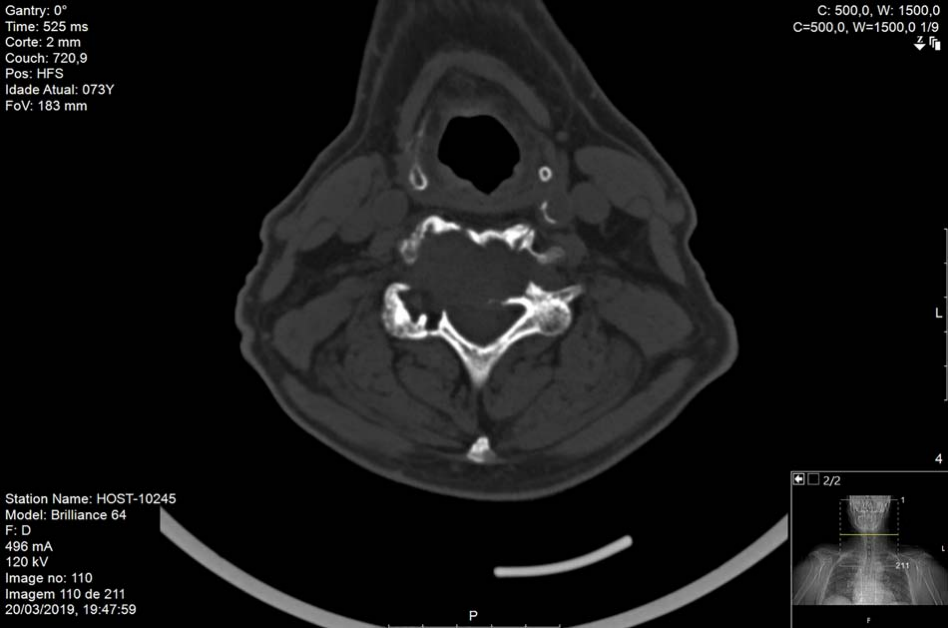
CT axial pre-RT.

**Figure 4:**
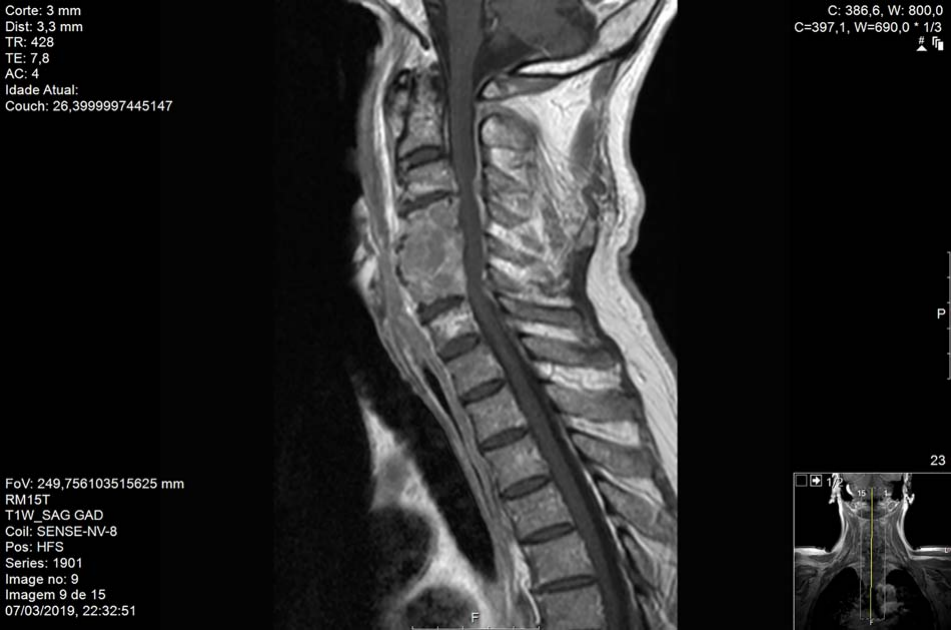
MR Sag T1 contrast pre-RT.

**Figure 5:**
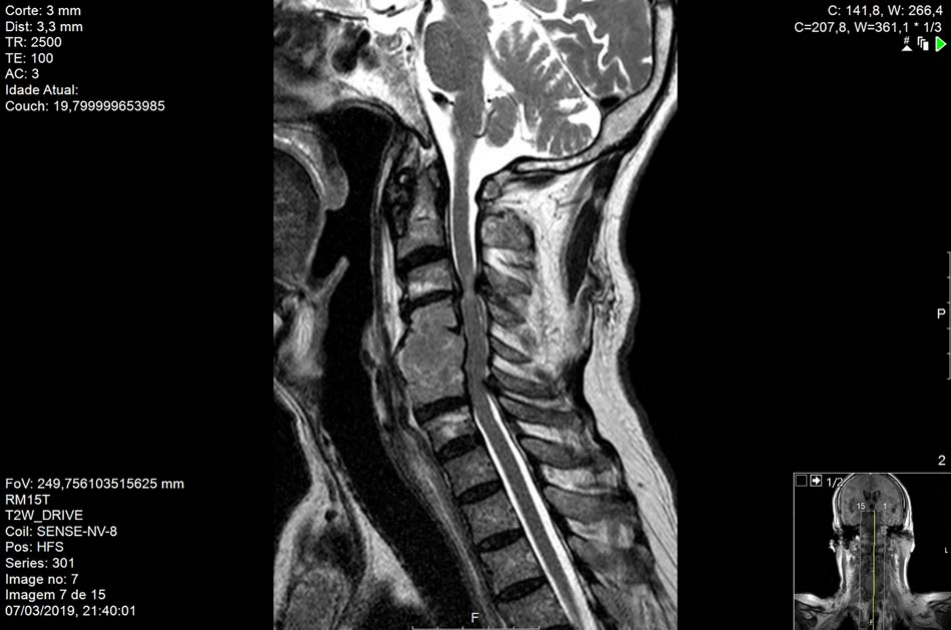
MR Sag T2 drive pre-RT.

**Figure 6:**
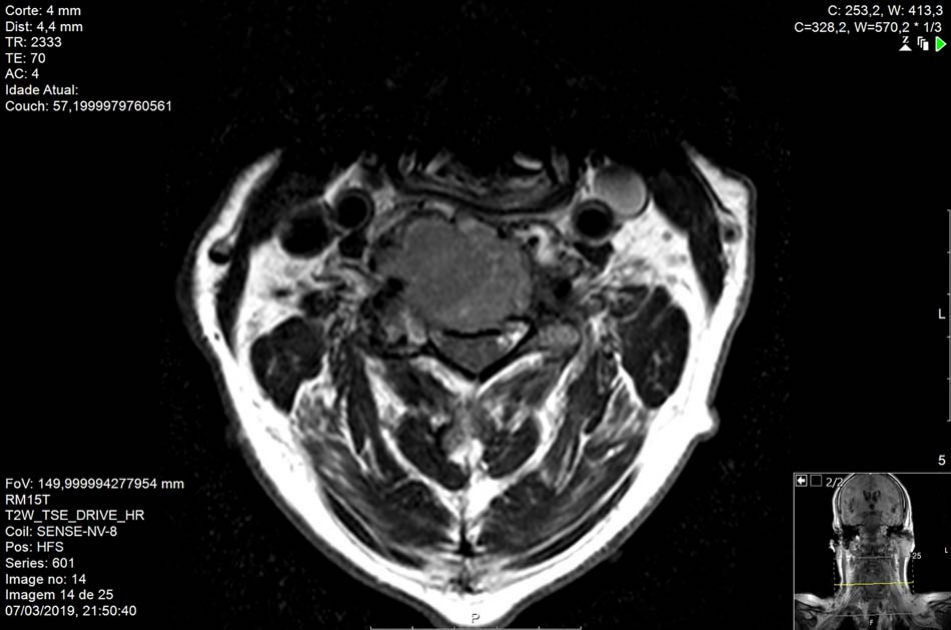
MR axial T2 pre-RT.

Computed tomography (CT) (Figs 1–3) and magnetic resonance imaging (MRI) (Figs 4–6) of the cervical spine were performed and revealed a lytic lesion involving most of C4, C5 and C6 vertebral bodies with bilateral extension to the posterior spinal elements of C4 and C5 and complete disruption of C4-C5 and C5-C6 intervertebral discs.

The Spinal Instability Neoplastic Score (SINS) [4, 6, 7] for assessing spinal instability from metastatic disease was used and the lesion was deemed unstable (SINS 13), with impending risk of increased neurological damage.

Findings from laboratory tests (serum protein electrophoresis) led to the diagnosis of MM. The patient was placed on a Philadelphia c-spine collar and underwent local 3D external-beam radiotherapy (20Gy in five fractions).

He was discharged at day 12 post-admission, pain-free and C5 motor deficit (motor strength grade IV).

At 3- and 6-month follow up the patient was pain-free, with no neurological deficits.

**Figure 7:**
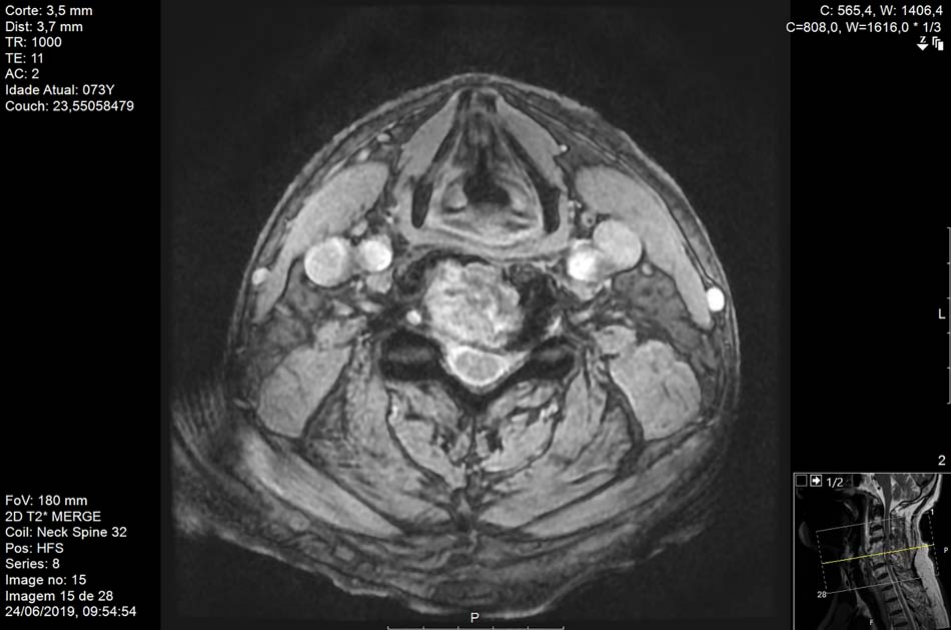
MR axial T2 Day 90 post-RT.

**Figure 8:**
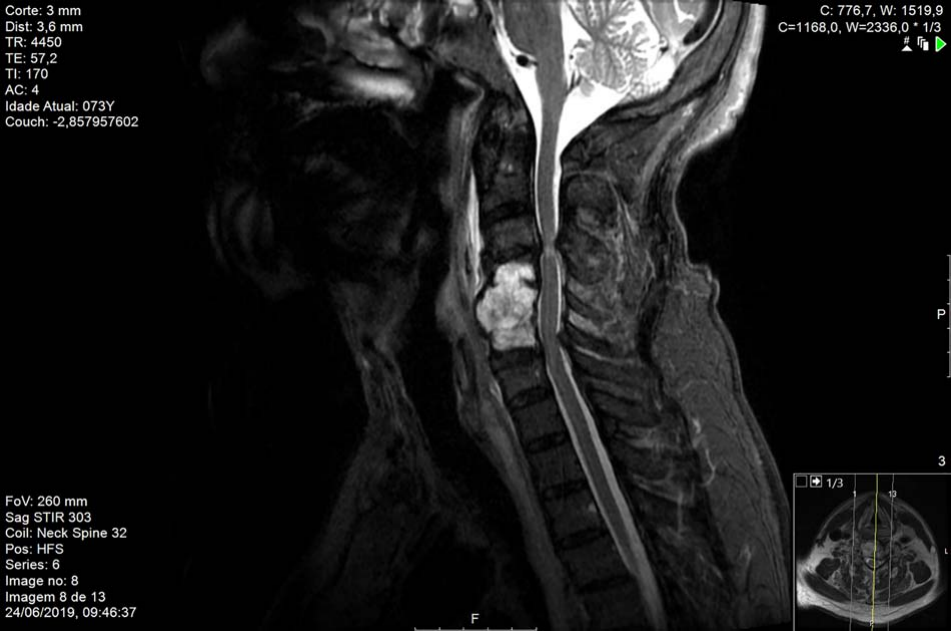
MR sagittal STIR Day 90 post-RT.

**Figure 9:**
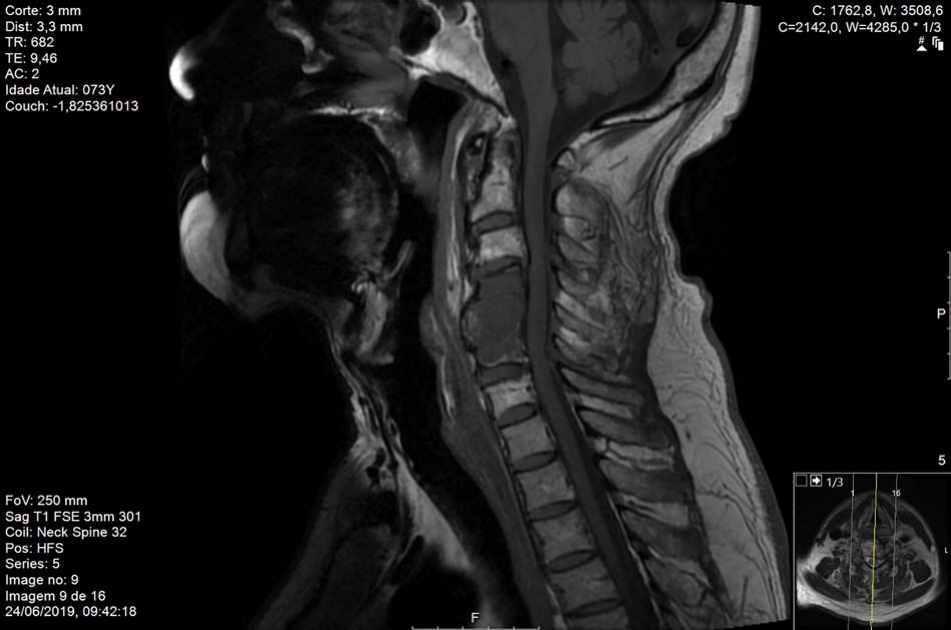
MR sagittal T1 Day 90 post-RT.

**Figure 10:**
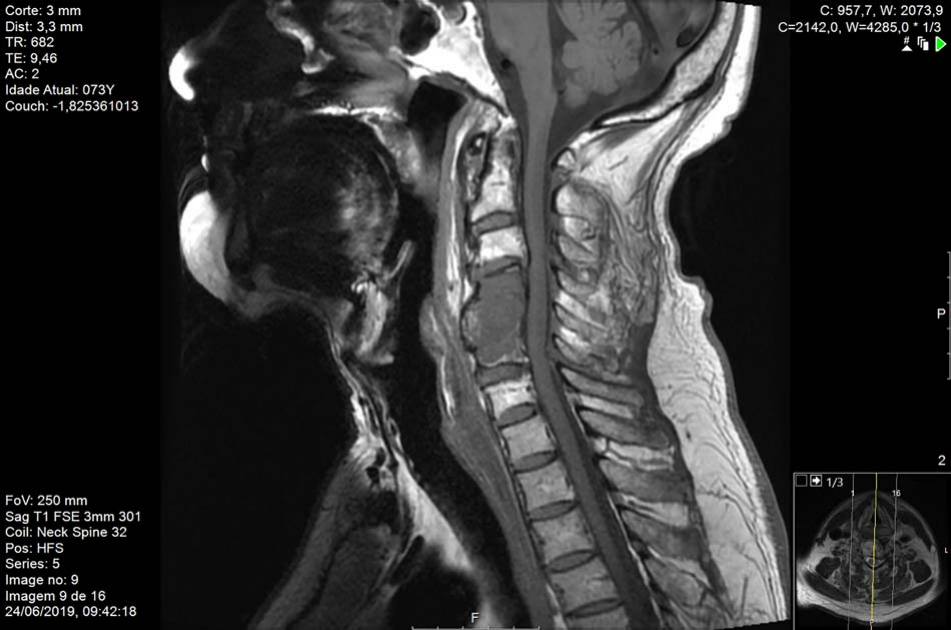
MR sagittal Day 90 post-RT.

**Figure 11:**
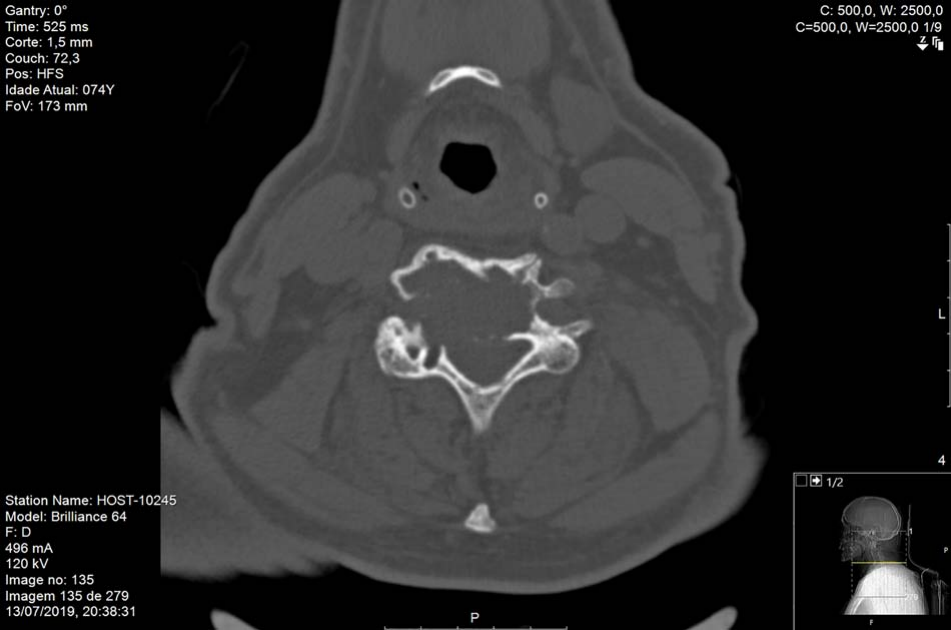
CT axial D120 post-RT.

**Figure 12:**
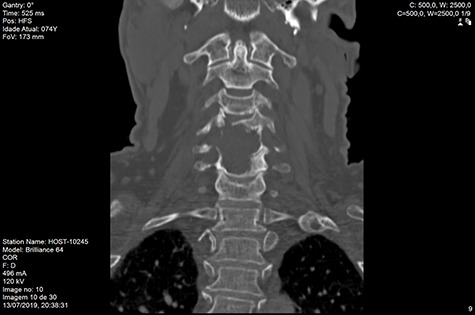
CT coronal D120 post-RT.

**Figure 13:**
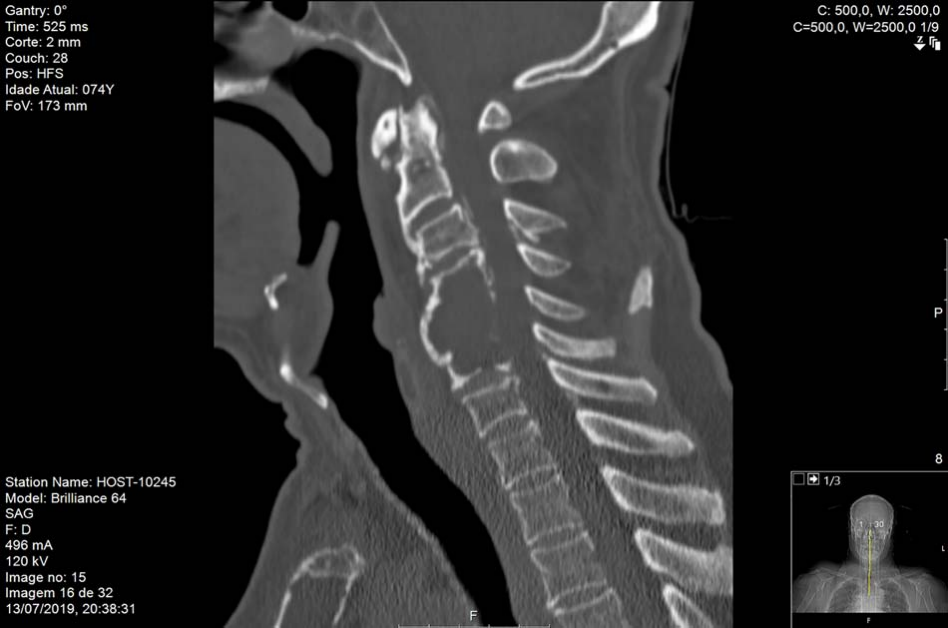
CT sagittal D120 post-RT.

MRI and CT scans performed at 90 days post-radiation therapy showed an arrest of further progression of instability and resolution of the lytic lesion (Figs 7–13).

## DISCUSSION

Discussion still ensues whenever cervical spinal metastasis from MM arise, specifically in cases with radiographically documented instability [1].

Unlike other spinal metastases, which may replace the marrow spaces of bone, multiple myeloma is characterized by active bone resorption.

As a result, spinal metastases from multiple myeloma may be considered more destructive, with spinal compression fractures occurring in up to 55–70% of patients with multiple myeloma.

Commonly, these fractures appear unstable and may prompt surgical intervention.

However, myeloma metastases should be approached with caution as the failure of anterior constructs for multiple myeloma of the cervical spine has been reported before, and is likely due to progression of the disease at adjacent levels and/or hardware loosening due to the poor quality of myelomatous bone.

None of the published cases of patients treated with radiation alone required further intervention after completing radiotherapy. It should be noted, however, that some of these patients died from progressive systemic disease soon after treatment for their cervical spine disease.

The reported outcomes of surgical stabilization for spinal instability are usually based on reviews of a variety of systemic malignancies.

It has become increasingly clear that multiple myeloma must be considered as a separate entity altogether. The diffuse nature of the disease, its impact on bone quality, and the resultant concerns for the failure of spinal fixation was in fact what led us to use radiation therapy and collar immobilization in patients who ultimately experienced healing of their fractures.

Although cases need to be discussed individually and surgery considered in cases of significant spinal instability or deformity and potential neurological injury, our results on this case supports the fact that isolated radiotherapy should be considered and be used to treat even extensively destructive osteolytic MM lesions to restore the structural integrity of the spinal elements.
